# Development and validation of the ID-EC - the ITALIAN version of the identify chronic migraine

**DOI:** 10.1186/s10194-019-0966-3

**Published:** 2019-02-13

**Authors:** Simona Sacco, Silvia Benemei, Sabina Cevoli, Gianluca Coppola, Pietro Cortelli, Francesco De Cesaris, Roberto De Icco, Cristiano Maria De Marco, Cherubino Di Lorenzo, Pierangelo Geppetti, Alessia Manni, Andrea Negro, Raffaele Ornello, Giulia Pierangeli, Francesco Pierelli, Lanfranco Pellesi, Luigi Alberto Pini, Francesca Pistoia, Maria Pia Prudenzano, Antonio Russo, Grazia Sances, Valentina Taranta, Cristina Tassorelli, Gioacchino Tedeschi, Maria Trojano, Paolo Martelletti

**Affiliations:** 1 Department of Applied Clinical Sciences and Biotechnology, Section of Neurology, University of L’Aquila, L’Aquila, Italy; 2 Department of Health Sciences, Section of Clinical Pharmacology and Oncology, University of Florence, Florence, Italy; 3 IRCCS Institute of Neurological Science of Bologna, Bologna, Italy; 4 Department of Medico-Surgical Sciences and Biotechnologies, Sapienza University Polo Pontino, Latina, Italy; 5 Department of Biomedical and Neuromotor Sciences, University of Bologna, Bologna, Italy; 6 Headache Science Center, IRCCS C. Mondino Foundation, Pavia, Italy; 7 Department of Clinical and Molecular Medicine, Regional Referral Headache Centre, Sant’Andrea Hospital, Sapienza University, Rome, Italy; 8 Department of Basic Medical Sciences, Neurosciences and Sense Organs, University of Bari, Bari, Italy; 9 IRCCS NEUROMED, Pozzilli, IS Italy; 10 Headache and Drug Abuse Research Centre, Policlinico Hospital, University of Modena e Reggio Emilia, Modena, Italy; 11 Headache Center, Department of Medical, Surgical, Neurological, Metabolic and Aging Sciences, University of Campania “Luigi Vanvitelli”, Caserta, Italy; 12 Department of Brain and Behavioral Sciences, University of Pavia, Pavia, Italy

**Keywords:** Migraine, Chronic migraine, Diagnosis

## Abstract

**Background:**

Case-finding tools, such as the Identify Chronic Migraine (ID-CM) questionnaire, can improve detection of CM and alleviate its significant societal burden. We aimed to develop and validate the Italian version of the ID-CM (ID-EC) in paper and as a smart app version in a headache clinic-based setting.

**Methods:**

The study investigators translated and adapted to the Italian language the original ID-CM questionnaire (ID-EC) and further implemented it as a smart app. The ID-EC was tested in its paper and electronic version in consecutive patients referring to 9 Italian tertiary headache centers for their first in-person visit. The scoring algorithm of the ID-EC paper version was applied by the study investigators (case-finding) and by patients (self-diagnosis), while the smart app provided to patients automatically the diagnosis. Diagnostic accuracy of the ID-EC was assessed by matching the questionnaire results with the interview-based diagnoses performed by the headache specialists during the visit according to the criteria of International Classification of Headache Disorders, III edition, beta version.

**Results:**

We enrolled 531 patients in the test of the paper version of ID-EC and 427 in the validation study of the smart app. According to the clinical diagnosis 209 patients had CM in the paper version study and 202 had CM in the smart app study. 79.5% of patients returned valid paper questionnaires, while 100% of patients returned valid and complete smart app questionnaires. The paper questionnaire had a 81.5% sensitivity and a 81.1% specificity for case-finding and a 30.7% sensitivity and 90.7% specificity for self-diagnosis, while the smart app had a 64.9% sensitivity and 90.2% specificity.

**Conclusions:**

Our data suggest that the ID-EC, developed and validated in tertiary headache centers, is a valid case-finding tool for CM, with sensitivity and specificity values above 80% in paper form, while the ID-EC smart app is more useful to exclude CM diagnosis in case of a negative result. Further studies are warranted to assess the diagnostic accuracy of the ID-EC in general practice and population-based settings.

## Introduction

Chronic migraine (CM) has an estimated prevalence of 2–3% in the general population [1, 2] and is associated with low health-related quality of life, significant loss of productive time and utilization of healthcare resources [3, 4], and a high prevalence of medication overuse [5]. Case-finding tools might improve the detection of CM, which remains underdiagnosed and undertreated worldwide despite its substantial burden [6]. Among those tools, the self-administered Identify Chronic Migraine (ID-CM) showed good diagnostic accuracy in a Web-based sample from a research panel [7]. The present study aimed to develop and validate an Italian version of the ID-CM, the *IDentificatore di Emicrania Cronica* (ID-EC; in English, ‘CM identifier’), in a paper form and in an electronic version as a ‘smart app’ in a nationwide, tertiary headache clinic-based setting. A secondary objective of the study was the assessment of acceptability of the tool by the patients.

## Materials and methods

**Fig. 1:**
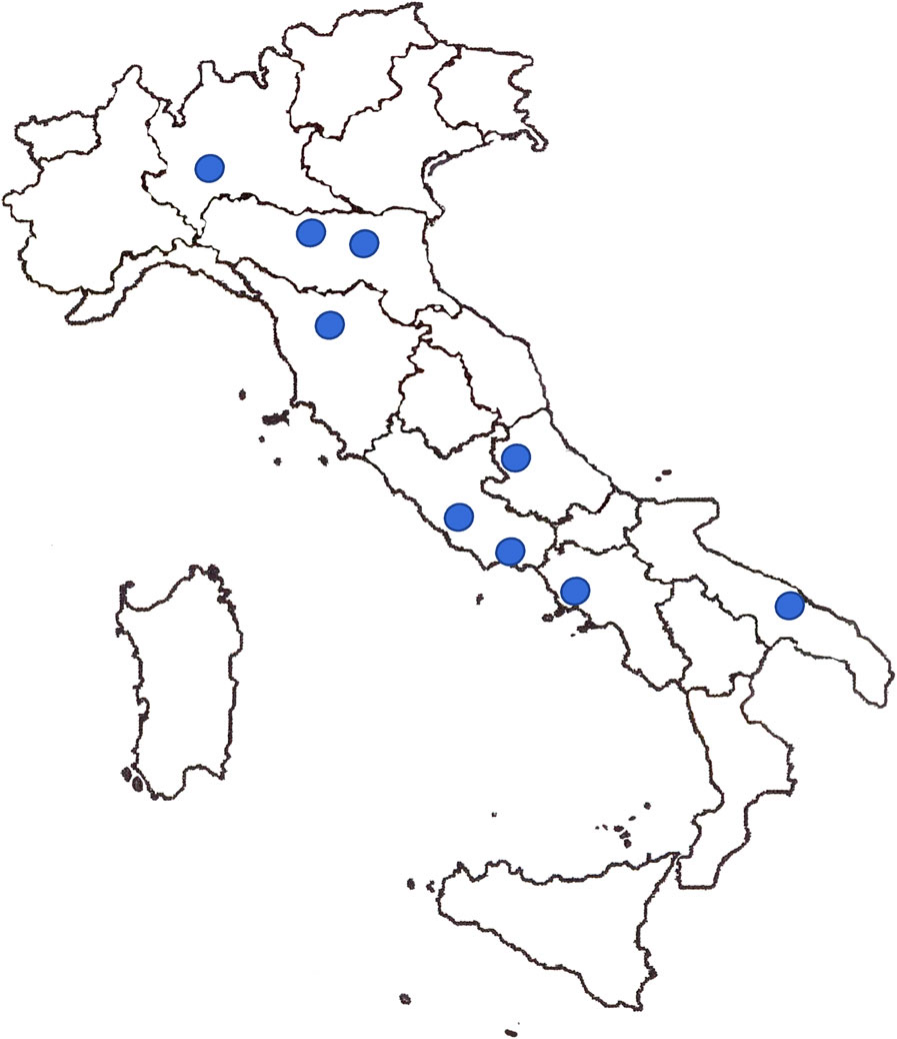
Map of the study centers

The study was approved by the local Ethics Committee of each of the 9 participating tertiary Headache Centers (Fig. 1). Each participant in the study signed an informed written consent.

### Development of the ID-EC

To develop the 12-item ID-EC, one study investigator (SS) translated the original version of the ID-CM into Italian. The translation was shared among all the study investigators during an in-person meeting; comments and suggestions which emerged during the meeting were considered to draft a revised version. The revised version was shared via e-mail among all members and further improved. The second draft was translated back into English, and compared it with the original English version to develop the final ID-EC.

The ID-EC smart app was developed from the same items of the paper form by a spin-off society, Smartly, and reviewed by all the study investigators. The smart app can be used on any Android or iOS electronic device and can be downloaded from the Apple Store or Google Play Store under the name ‘Rilevatore di Emicrania Cronica’. A link to use the electronic version on computers is available at https://fondazioneitalianastudiocefalee.it/rilevatore-di-emicrania-cronica/.

### Validation of the ID-EC

We included in the validation study all consecutive patients, aged 18 years or older, referring for the first time to the study centers. The cost of visits and treatments is covered, partially or totally, by the Italian National Health Service. Subjects were excluded for unwillingness to participate in the project, language barrier, or cognitive disturbances affecting the capacity of filling in the ID-EC questionnaire responses.

**Fig. 2:**
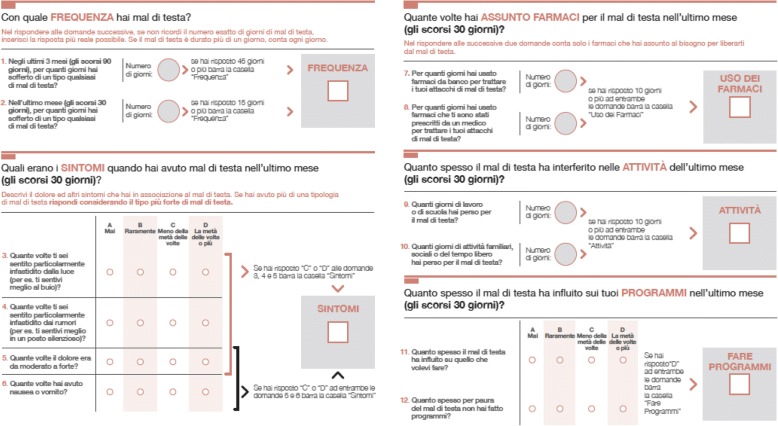
The IDentificatore di Emicrania Cronica (ID-EC)

For each patient, we recorded age, sex, and headache history, including frequency, duration, and previous clinical evaluations for headache. Before in-person visits, patients self-administered the ID-EC paper or smart app questionnaires (Fig. 2). Paper questionnaires were considered not valid and excluded from the study if unreadable or containing contradictory information. In the case of the smart app, answer input and questionnaire completion were guided. The paper questionnaire requires the application of a diagnostic algorithm to reach the diagnosis of possible CM [7]. In our study, this diagnostic algorithm was applied either by the study investigators (case-finding) or by patients (self-diagnosis). Conversely, in the case of the smart app questionnaire, the app automatically applied the scoring algorithm and provided a diagnosis without the need of applying the diagnostic algorithm either by investigators or by patients. After ID-EC completion, headache experts blinded to the questionnaire results assigned the clinical headache diagnoses according to the criteria of International Classification of Headache Disorders, III edition, beta version [8]; those diagnoses were used as the gold standard for ID-EC validation. All the data were collected using a Web-based form.

### Acceptability

After compiling the ID-EC paper or smart app questionnaires, patients were asked to compile an acceptability questionnaire containing 5 questions for the paper form and 8 questions (i.e. the same of the paper form plus 3 additional ones) for the smart app version (Table 1).

**Table 1:** English translation of the acceptability questionnaire

Common to the paper and smart app questionnaires	
Were the questions clear?	
Were the questions appropriate with respect to your clinical problem?	
Was the questionnaire complex?	
Did you need help to complete the questionnaire?	
Did you think the questions were confusing?	
Specific to the smart app questionnaire	
Are you at your ease in use of smartphones or tablets?	
Would have preferred a paper questionnaire?	
Were the questions easy to dial?	

### Statistical analysis

Descriptive statistics are presented as mean ± standard deviation, median with interquartile range, or absolute numbers and proportions, as appropriate. We calculated the sensitivity, specificity, negative predictive values (NPV), and positive predictive values (PPV) of ID-EC by matching clinical diagnoses with questionnaire data.

We estimated the study sample size using formulas that take into account a clinically acceptable degree of precision of the screening test, the hypothesized values of sensitivity and specificity, and the estimated prevalence of disease in the target population [9]. Assuming a maximum clinically acceptable 95% confidence interval width of 10%, an estimated CM prevalence of 30–50% in tertiary headache centers, and the 80.6% sensitivity and 88.6% specificity of the ID-CM [7], we deemed necessary a minimum sample size of 200 subjects with CM in each part of the validation study.

## Results

The paper form validation was performed from November 2017 to April 2018, while the smart app validation was performed from April to September 2018.

### Characteristics of patients

Table 1 reports the characteristics of the 532 subjects included in the paper questionnaire validation study and of the 427 subjects included in the smart app validation study. The two study populations had different distributions of clinical headache diagnoses (Table 2). In both study populations, the most prevalent disorder was episodic migraine with or without aura, followed by CM; the prevalence of medication overuse headache without CM, tension-type headache, other primary headaches, and secondary headaches was relatively low in both populations (Table 2).

**Table 2:** Clinical data of the included subjects

Characteristics	Paper version (*N* = 532)	Smart app version (*N* = 427)	*P* value
Female (no, %)	435 (81.2)	350 (82.0)	0.936
Age (mean ± SD)	42.0 ± 13.0	44.8 ± 14.1	
Years from headache onset (mean ± SD)	23.6 ± 14.4	26.7 ± 15.2	
Over-the-counter medication use (no, %)	466 (87.6)	336 (78.7)	< 0.001
Previous consultations for headache (no, %)	341 (64.1)	323 (75.6)	< 0.001
Previous specialist advice for treatment (no, %)	298 (56.0)	297 (69.6)	< 0.001
Clinical diagnoses (no, %)^a^			
Episodic migraine			
Without aura	260 (48.9)	177 (41.5)	0.022
With aura	48 (9.0)	33 (7.7)	0.474
Chronic migraine	209 (39.3)	202 (47.3)	0.013
Episodic tension-type headache	17 (3.2)	18 (4.2)	0.403
Chronic tension-type headache	20 (3.8)	5 (1.2)	0.012
Other primary headache	41 (7.7)	20 (4.7)	0.057
Secondary headache	12 (2.3)	5 (1.2)	0.206

^a^results may add up to over 100% due to possible comorbidities

### Diagnostic accuracy

**Fig. 3:**
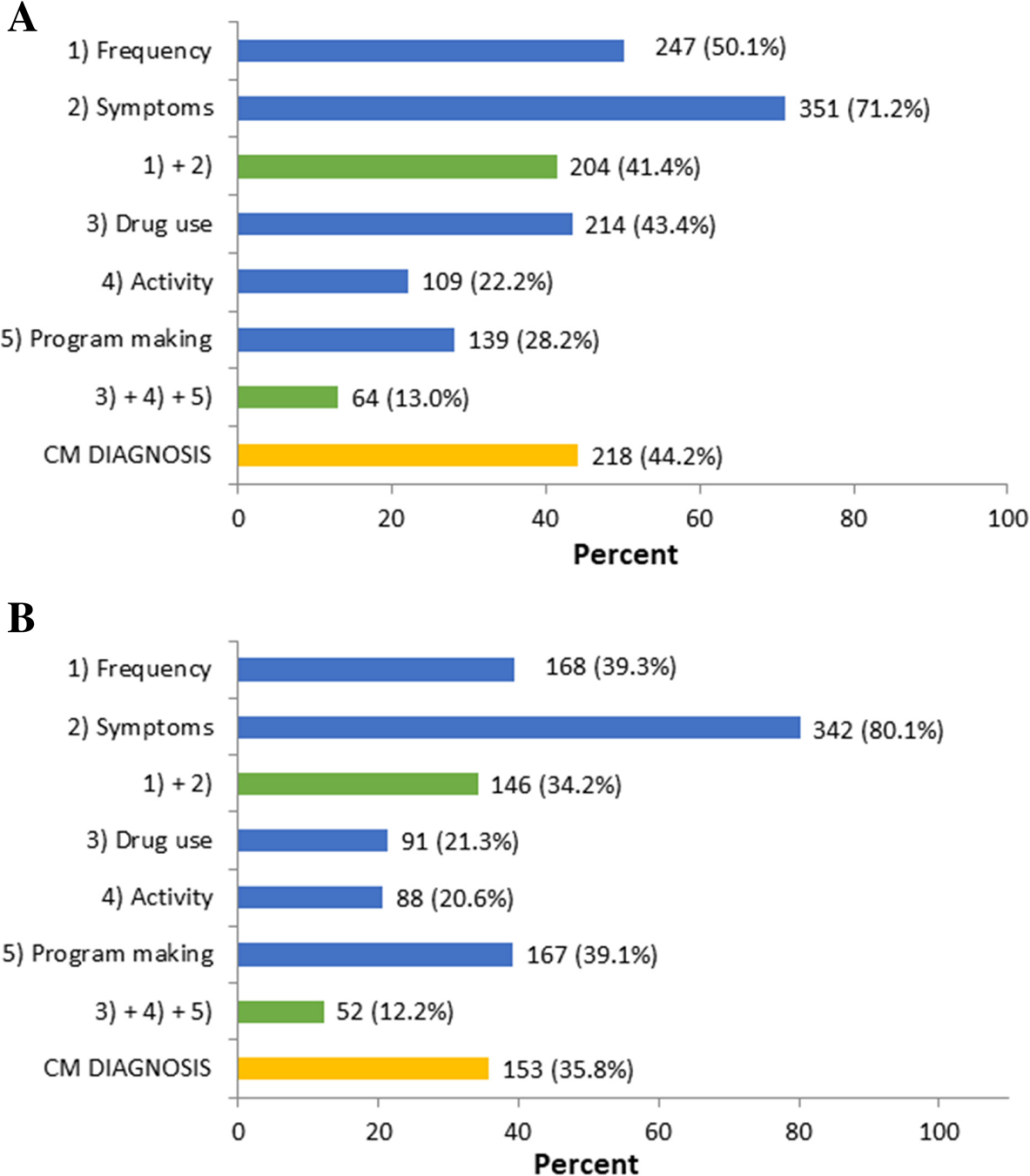
Proportions of boxes checked in the ID-EC paper (**a**) and smart app (**b**) questionnaires

Among the 532 subjects included in the paper questionnaire validation study, 1 refused to complete the questionnaire and 38 had an invalid questionnaire, while all the 427 subjects included in smart app validation study had valid and complete questionnaires. According to the ID-EC, among the 493 paper version usable questionnaire, 218 (44.2%) met criteria for possible CM, while among the 427 smart app questionnaires, 153 (35.8%) met criteria for possible CM (Table 3). Figure 3 reports the proportions of checked ID-EC boxes; notably, in both the paper form and the smart app questionnaire the first two boxes were checked by higher proportions of patients compared with the last three boxes (Fig. 3).

**Table 3:** Data matching of questionnaires and clinical diagnoses

No. of patients	Chronic migraine(clinical)	No Chronic migraine (clinical)	Total
Paper version			
Chronic migraine	150	68	218
No Chronic migraine	35	240	275
Total	185	308	493
Smart app version			
Chronic migraine	131	22	153
No Chronic migraine	71	203	274
Total	202	225	427

The paper questionnaire had a 81.5% sensitivity, a 81.1% specificity, a NPV of 72.3%, and a PPV of 88.8% for case-finding, based on the CM prevalence of 39.3% in the headache centers (Table 4). The corresponding values for self-diagnosis were 30.7%, 95.2%, 81.0%, and 67.3%, respectively (Table 4). Conversely, the smart app had a 64.9% sensitivity, a 90.2% specificity, a NPV of 74.1%, and a PPV of 85.6%, based on the CM prevalence of 47.3% in headache centers (Table 3). Among false positive patients, the most common misdiagnoses were episodic migraine without aura in both the paper (39.7%) and smart app (52.4%) questionnaires (Table 5).

**Table 4:** Diagnostic accuracy of the ID-EC paper and smart app questionnaires

	Sensitivity (%)	Specificity (%)	NPV (%)^a^	PPV (%)^a^
Paper version	81.5	81.1	72.3	88.0
Smart app version	64.9	90.2	74.1	85.6
English version [7]	80.6	88.6	75.0	91.5

*NPV* indicates negative predictive value, *PPV* positive predictive value

^a^assuming a prevalence of CM of 39.3% in paper versions and 47.3% in smart app validation

**Table 5:** Clinical diagnosis distribution of false positive patients

Clinical diagnosis (no, %)	Paper version (*N* = 68)	Smart app version (*N* = 22)
Episodic migraine		
Without aura	27 (39.7)	11 (50.0)
With aura	3 (4.4)	–
Medication overuse headache	14 (20.6)	4 (18.2)
Episodic tension-type headache	15 (22.1)	–
Chronic tension-type headache	6 (8.8)	–
Other primary headache	3 (4.4)	7 (31.8)

As some of the centers did not participate to the paper form questionnaire validation, we repeated the analyses for the smart app only including the centers which also participated to the paper form validation; in the 418 remaining patients, the smart app had a 65.5% sensitivity, a 90.4% specificity, a NPV of 74.5% and a PPV of 85.9%.

### Acceptability

**Fig. 4:**
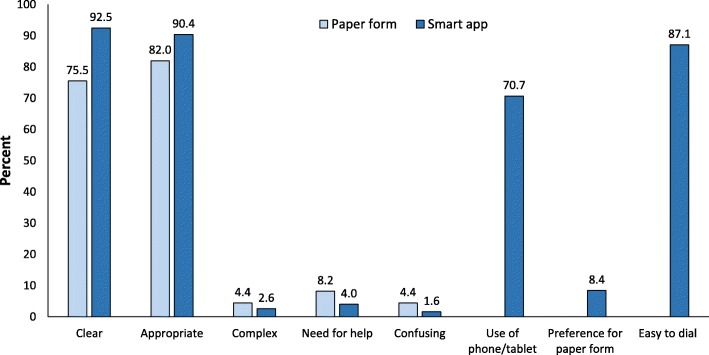
Results of acceptability questionnaires for the ID-EC paper and smart app questionnaires. Proportions refer to affirmative answers

Acceptability questionnaires for the paper and for the smart app questionnaires were returned by 502 (94.5%) and 425 (99.5%) patients, respectively. In both studies, most subjects (> 90%) found the questions clear and appropriate, while only a small proportion of patients (< 10%) reported them as complex or confusing and admitted to have required help for answering (Fig. 4). Furthermore, most patients (> 90%) in the smart app study found it easy to type the answers, would not have preferred a paper questionnaire, and declared themselves familiar with the use of smartphones and tablets (Fig. 4).

## Discussion

In the present study, we developed a paper and a smart app version of the ID-EC, the Italian version of ID-CM. Besides, our study proved the applicability of the CM case-finding tool on widely available technological supports, including computers, smartphones, and tablets. The case-finding sensitivity and specificity of the paper questionnaire were higher than 80%, in line with the original ID-CM [7]. The smart app version had a lower but acceptable sensitivity paralleled by a higher specificity; thus, the smart app version works better to exclude CM diagnosis in case of a negative result. The different diagnostic accuracy of the paper questionnaire compared with the smart app might be attributed to the different composition of the study populations, as the paper questionnaire validation study population had a higher prevalence of CM and a lower proportion of patients without previous headache referral or treatment compared with the smart app study population. Notably, the ‘frequency’ box of the questionnaire was checked by a higher proportion of patients in the paper questionnaire of the ID-EC compared with the smart app, which might also have influenced the test sensitivity. Besides, some of the study centers participated in the smart app but not in the paper questionnaire validation study because of delayed approval from Ethics Committees; however, after the exclusion of the patients included by the centers with delayed approval from Ethics Committees, the smart app validation results remained the same, possibly because of the limited number of excluded patients. The different volumes of activity of the study centers and seasonal variations might also have influenced the study results.

The relatively low sensitivity of CM self-diagnosis in our study suggests that the paper form of the ID-EC should be limited to clinical settings, where the diagnostic algorithm can be checked by trained personnel. Conversely, the smart app version might be suitable as a self-diagnostic tool in the general population without any specific support, as it performs an accurate automatic scoring which considerably improves self-diagnosis. In fact, also the self-diagnostic accuracy of the paper questionnaire might be increased by providing cues for self-application of the diagnostic algorithm and making the questionnaire completion mandatory.

Both the paper and the smart app questionnaires may improve CM detection by physicians not familiar with CM diagnosis, thus contributing to overcome the barriers to CM care. Indeed, data from the American Migraine Prevalence and Prevention (AMPP) Study showed that only 56% of participants meeting the diagnostic criteria for migraine had ever received a migraine diagnosis from a health care professional [10], and the percentage was even lower (20%) when CM diagnosis was considered [10]. Besides, data from the longitudinal Chronic Migraine Epidemiology and Outcomes (CaMEO) Study [11] proved that only a minority of participants with CM (13.6%) reported consulting a specialist for diagnosis and treatment of migraine [12], and even among patients with CM who consulted a specialist, only 36% reported receiving an appropriate diagnosis [12]. A multicenter study indicated that also in Italy, migraine is underdiagnosed and undertreated [13]. In fact, the diagnosis of migraine is missed even when patients seek for medical advice, mostly when referring to general practitioners. Enhancing CM detection is relevant because CM imposes a substantial burden on individuals, families and society. Indeed, the International Burden of Migraine Study (IBMS) reported the mean headache-related direct costs over a 3-month period for individuals in United States with costs for those with CM were notably higher at $1036/person [14]. These findings are consistent with those from the AMPP, which reported per person annual total cost for CM of $1757 [15]. According to IBMS Study, in Italy the annual individual cost of CM is more than 3 times higher than that of EM (€ 2669.80 vs € 828.52) [16].

The study has some limitations. The studied population largely differs from the general population, because of a high CM prevalence and a long history of headache, with many consultations and treatment failures before first referral to tertiary headache centers. We chose to develop and validate the ID-EC in those centers because they are held by headache experts able to develop diagnostic tools according to their wide clinical knowledge; however, the ID-EC is intended for use in general practices, where headache experts are lacking and the prevalence of CM is lower compared with tertiary headache centers. In general practices, physicians might be less accurate in data collection compared with headache experts; however, the use of automatic scoring provided by smart app might help non-expert physicians to select their patients for appropriate referral. Besides, patients in general practices are more frequently treatment-naïve compared with those referring to tertiary headache centers; therefore, despite a lower CM prevalence, they might self-diagnose CM with reasonable accuracy, as they are less influenced by previous prescriptions. To test those hypotheses, further studies are needed in general practices to improve the identification and management of CM patients.

## Conclusions

Our findings suggest the validity and acceptability of the ID-EC, the Italian version of the ID-CM developed as a paper and a smart app questionnaires, as a case-finding tool for CM. Our study prompts the assessment of the diagnostic accuracy of the tool in general practices. The high acceptability of ID-EC and the availability of a smart app warrant its potential large-scale implementation.

## Abbreviations

AMPP: American Migraine Prevalence and Prevention
CaMEO: Chronic Migraine Epidemiology and Outcomes
CM: Chronic migraine
IBMS: International Burden of Migraine Study
ID-CM: Identify Chronic Migraine
ID-EC: IDentificatore di Emicrania Cronica
NPV: Negative predictive value
PPV: Positive predictive value
